# Purine Nucleoside Phosphorylase Deficiency: A Case Report of an Extremely Rare Disorder

**DOI:** 10.7759/cureus.76220

**Published:** 2024-12-22

**Authors:** Badriah G Alasmari, Fawzy Ibrahim, Shady Wafa, Abdullah Alshehri, Muhammad Saeed

**Affiliations:** 1 Pediatrics, Armed Forces Hospital Southern Region, Khamis Mushayt, SAU; 2 Pediatrics Critical Care Unit, Armed Forces Hospital Southern Region, Khamis Mushayt, SAU; 3 Pediatric Neurology, Armed Forces Hospital Southern Region, Khamis Mushayt, SAU

**Keywords:** hematopoietic stem cell transplant, neurologic manifestation, neutrophil count, purine nucleoside phosphorylase deficiency, severe combined immunodeficiency (scid)

## Abstract

Purine nucleoside phosphorylase (PNP) deficiency is one of the very rare types of immune deficiency disorders inherited in an autosomal recessive (AR) manner. PNP deficiency is a progressive immune disorder that can range from severe combined immunodeficiency (SCID) to combined immunodeficiency and is associated with recurrent infections, neurological manifestations, and sometimes autoimmune disorders. In our case, we describe the case of a female patient, two years and six months old, with recurrent infections, severe neutropenia, failure to thrive, and a history of a deceased sister with the same condition. She was diagnosed with PNP through genetic testing, which confirmed the homozygous variant c.46T>C p.(Trp16Arg) in PNP, in addition to clinical manifestations and the positive history of her sister.

## Introduction

Purine nucleoside phosphorylase (PNP) deficiency is the enzyme of purine metabolism that functions in the salvage pathway. This allows cells to utilize purine bases to synthesize purine nucleotides. Giblett et al. first described PNP deficiency in 1975 after discovering adenosine deaminase (ADA) deficiency. Both are important enzymes in the purine metabolic pathway [1]. Decreased or absent PNP function leads to deoxyguanosine triphosphate (dGTP) accumulation in the mitochondria, causing thymus selection apoptosis, DNA damage, and T lymphocyte dysfunction. PNP deficiency is a very rare primary immunodeficiency disorder (less than 5%) inherited by autosomal recessive (AR) manner that leads to an impairment of T-cell function with sometimes B-cell function impairment. PNP is characterized by three main manifestations of neurological dysfunctions such as ataxia, intellectual disability and developmental delay, recurrent infection, and autoimmunity [2]. PNP deficiency disorder can present with different abnormalities, but neurological symptoms are among the most common and affect about 70% of individuals. Autoimmune disorders are present in about 30% of patients, with autoimmune hemolytic anemia being the most common presentation, followed by immune thrombocytopenia and autoimmune neutropenia. Hematological malignancies may occasionally occur [3]. PNP can be discovered in newborn screening through the use of liquid chromatography-tandem mass spectrometry. The only curative treatment for PNP is hematopoietic stem cell transplantation (HSCT) [4].

Here, we present a new case of a female patient from the southern region of Saudi Arabia who presented with recurrent infections, global developmental delay, and central hypotonia.

## Case presentation

We present a case of two years and six-month-old female child delivered to a consanguineous marriage. She was late preterm 36+3 weeks, delivered by cesarean section due to decreased end diastolic flow then admitted to NICU for four months due to low birth weight, and intrauterine growth restriction (IUGR). There was no delayed cord separation or other systemic abnormalities. She was the second baby with an older sibling who died with the same condition at the age of one year. The condition started at four months of age in the NICU, when routine lab tests showed severe neutropenia, leukopenia, and lymphopenia. These findings, along with her sister's medical history, raised suspicion of an immunodeficiency disorder. Therefore, an immunoglobulin assay was performed, and the results were normal, bone marrow aspiration was also performed, which showed reactive bone marrow with no evidence of leukemia or lymphoma. So, the patient was discharged from NICU with follow-up in our hematology clinic and was managed by subcutaneous granulocyte-colony stimulating factor (GCSF) weekly. However, the patient had recurrent hospital admissions for bronchiolitis and gastroenteritis. During these admissions, the patient received GCSF in addition to her weekly dose, but she still had persistent leukopenia throughout all admissions. At the age of 31 months, the patient came to our hospital with lethargy, severe dehydration due to vomiting and diarrhea for three weeks, severe oral thrush, unilateral neck abscess, and scalp abscess. So the patient was admitted with a case of gastroenteritis and septic shock, and full septic screening was done (Table 1) including abscess aspiration and culture, and received wide-spectrum antibiotics. Abscess culture showed Methicillin-resistant Staphylococcus aureus (MRSA) blood, and urine culture showed no growth. Also, a whole exomes sequence (WES) study and total complement activity (CH50) were done. After completing the treatment with antibiotics with supportive measures, the patient was discharged home in good condition with stable vital signs and no more pictures of infection or dehydration. Unfortunately, two days after discharge, the patient was brought by her family to the emergency room (ER) dead on arrival. The cause of death was unclear, and the family did not provide a clear history. Later, the WES result came back showing the homozygous variant c.46T>C p. (Trp16Arg) in PNP (OMIM:164050), which leads to an amino acid exchange. The total complement activity (CH50) result was less than 10 U/mL (reference range 32-58 U/mL).

**Table 1:** Hematological and biochemical investigations IgG: immunoglobulin G; IgA: immunoglobins A; IgM: immunoglobins M

Lab test	Lab result		Reference range
Hemoglobin	9.6 g/dL		10-14 g/dL
Total WBC	1.31×10^9^/L		6-17×10^9^/L
Platelets	397×10^9^/L		150-450×10^9^/L
Absolute neutrophils	0.30×10^9^/L		1.8-8×10^9^/L
Absolute lymphocytes	0.39×10^9^/L		2.5-16.5×10^9^/L
IgG	10.70 g/L		7.51-15.6 g/L
IgA	1.25 g/L		0.82-4.53 g/L
IgM	0.60 g/L		0.46-3.04 g/L

## Discussion

PNP deficiency was first described in 1975. A few cases, mostly from the Middle East, have been reported. PNP deficiency accounts for less than 5% of all severe combined immunodeficiency (SCID) cases worldwide [5]. PNP is a progressive immune abnormality that can range from SCID to combined immunodeficiency and is associated with recurrent infections, neurological manifestations, and sometimes autoimmune disorders. Neurological dysfunction, immune deficiency, or autoimmunity are required to suspect the diagnosis. Genetic analysis like a WES study is essential for the diagnosis. We reported a case of PNP. This patient had a history of an older sibling who died from the same condition and presented with severe neutropenia, leukopenia, and lymphopenia, which appeared at the age of four months. The patient had subsequent recurrent hospital admissions due to infections, including oral thrush, gastroenteritis, and acute bronchiolitis. All of these presentations are consistent with immune deficiency. Additionally, our patient presented with global developmental delay and neurological problems, including central hypotonia.

A study was published reporting on four patients diagnosed with PNP deficiency, two of whom were diagnosed before the age of one. The patients presented with lymphopenia, reduced uric acid levels, and neurologic findings, a clinical picture of severe combined immune deficiency. Genetic studies were performed, and a definite diagnosis of PNP deficiency was made. All patients presented with almost the same presentation of recurrent infection with acute bronchiolitis, pneumonia, and moniliasis, and all of them with neurological disorders vary from mental and motor retardation to microcephaly and convulsions. Additionally, all patients had a family history of consanguinity between their parents. Unfortunately, all patients were lost at the age between seven months and forty-seven months [6]. Another study also mentioned six Egyptian patients with PNP deficiency from different families with positive consanguinity in three of them. The study mentioned positive history in those families with the same condition and also dead siblings. The clinical findings in these patients regarding infections were the same as SCID presentations in the form of recurrent pneumonia, chronic diarrhea, and oral thrush. The study mentioned that five of these patients presented with leukopenia and immune cytopenia in the form of autoimmune hemolytic anemia, and they required frequent blood transfusions. Neurological manifestations were also found in the form of developmental delay, hypotonia, microcephaly, and delayed milestones [7]. A case scenario reported a 33-month-old, full-term female Saudi child with first-degree consanguinity and AR PNP deficiency. The patient presented with global developmental delay, central hypotonia, and a history of recurrent oral thrush, respiratory infections, and perianal abscess [8]. Compared with other studies and reports on PNP, our patient is similar to these reports in terms of positive consanguinity, recurrent infections, and neurological disorders. Most of the reported cases have had similar outcomes to our patients.

A multidisciplinary specialties team is required for the diagnosis and treatment of PNP deficiency, recognizing clinical signs and symptoms with active action plans for evaluations and diagnosis to prevent complications. The only cure for many immunodeficiencies, including PNP deficiency, is HSCT. Gene therapy could be a better treatment option, as it does not require a donor, such as first-generation γ-retroviral vectors or more recent lentiviral vectors. Until then, patients with PNP deficiency should be isolated at home and minimize the risk of infection by regular hand washing, avoiding visitors, and taking other protective measures. If the patient is admitted to the hospital, they should also be isolated. Patients with PNP deficiency die rapidly, if left untreated [8].

## Conclusions

PNP is a rare immunodeficiency disorder that should be considered in cases of recurrent infections, neurological manifestations, and autoimmune disorders. In this case report, we present a typical clinical picture of PNP deficiency to raise awareness of the disease, as early diagnosis can aid in a favorable prognosis. If left untreated, PNP deficiency rapidly causes death due to recurrent infections, so diagnosis should be established as early as possible. Prenatal diagnosis is currently available. HSCT and gene therapy could be better treatment options.
